# Extensive chemical and bioassay analysis of polycyclic aromatic compounds in a creosote-contaminated superfund soil following steam enhanced extraction^☆^

**DOI:** 10.1016/j.envpol.2022.120014

**Published:** 2022-08-23

**Authors:** Ivan A. Titaley, Lisandra Santiago Delgado Trine, Thanh Wang, Daniel Duberg, Eva L. Davis, Magnus Engwall, Staci L. Massey Simonich, Maria Larsson

**Affiliations:** a Man-Technology-Environment (MTM) Research Centre, School of Science and Technology, Örebro University, Örebro SE-701 82, Sweden; b Department of Environmental and Molecular Toxicology, Oregon State University, Corvallis, OR, 97331, USA; c Center for Environmental Solutions & Emergency Response, Groundwater, Watershed and Ecosystems Restoration Division, United States Environmental Protection Agency, Ada, OK, 74820, USA; d Department of Chemistry, Oregon State University, Corvallis, OR, 97331, USA

**Keywords:** Polycyclic aromatic compounds, Estrogen receptor, Androgen receptor, Aryl hydrocarbon receptor, Mass defect, Remediation

## Abstract

Polycyclic aromatic compounds (PACs) are organic compounds commonly found in contaminated soil. Previous studies have shown the removal of polycyclic aromatic hydrocarbons (PAHs) in creosote-contaminated soils during steam enhanced extraction (SEE). However, less is known about the removal of alkyl-PAHs and heterocyclic compounds, such as azaarenes, and oxygen- and sulfur-heterocyclic PACs (OPACs and PASHs, respectively). Further, the impact of SEE on the freely dissolved concentration of PACs in soil as well as the soil bioactivity pre- and post-SEE have yet to be addressed. To fulfil these research gaps, chemical and bioanalytical analysis of a creosote-contaminated soil, collected from a U.S. Superfund site, pre- and post-SEE were performed. The decrease of 64 PACs (5–100%) and increase in the concentrations of nine oxygenated-PAHs (OPAHs) (150%) during SEE, some of which are known to be toxic and can potentially contaminate ground water, were observed. The freely dissolved concentrations of PACs in soil were assessed using polyoxymethylene (POM) strips and the concentrations of 66 PACs decreased post-SEE (1–100%). Three *in vitro* reporter gene bioassays (DR-CALUX^®^, ERα-CALUX^®^ and anti-AR CALUX^®^) were used to measure soil bioactivities pre- and post-SEE and all reporter gene bioassays measured soil bioactivity decreases post-SEE. Mass defect suspect screening tentatively identified 27 unique isomers of azaarenes and OPAC in the soil. As a remediation technique, SEE was found to remove alkyl-PAHs and heterocyclic PACs, reduce the concentrations of freely dissolved PACs, and decrease soil bioactivities.

## 1. Introduction

Soil remediation plays an integral role in achieving a healthy ecosystem. The United Nations has laid out 17 Sustainable Development Goals (UN SDGs) (Rosa, 2017) and ‘Life on land’ is listed as one of the UN SDGs, of which remediation of contaminated soil is considered an integral component in achieving this goal (Keesstra et al., 2018). Polycyclic aromatic compounds (PACs) are group of compounds commonly found in contaminated soil. This group of contaminants encompasses a variety of compounds, including unsubstituted (parent) polycyclic aromatic hydrocarbons (PAHs), 16 of which are listed in the U.S. Environmental Protection Agency priority pollutants (PAH 16), alkylated-PAHs (alkyl-PAHs), PAHs with molecular weight 302 u (MW302-PAHs), and oxygenated-PAHs (OPAHs). Heterocyclic compounds, such as N-, S-, and O-heterocyclics (azaarenes, PASHs, and OPACs, respectively), are also examples of PACs. In this work, OPAHs refer to compounds such as 9-fluorenone, while OPACs refer to compounds such as benzo [*b*]naphtho [2,1-*d*]furan. The carcinogenicity of some PAHs are known (IARC, 2010), but some alkyl-PAHs are more bioactive in estrogen receptor (ER) and in aryl hydrocarbon receptor (AhR) based *in vitro* assays than their corresponding parent compounds (Lam et al., 2018b, 2018a), while some MW302-PAHs are known to be carcinogenic and AhR active (Long et al., 2016; Vondráček et al., 2020). Compounds such as OPAHs are also known toxicants (Geier et al., 2018; Knecht et al., 2013). Meanwhile, some azaarenes have shown carcinogenic and mutagenic effects and are known to be bioactive *via* ER or AhR pathway (Bleeker et al., 2002; Hinger et al., 2011; Machala et al., 2001). Both PASHs and OPACs are less studied, but some are found to be bioactive (Brinkmann et al., 2014; Hinger et al., 2011). Due to the toxicity, carcinogenicity, and bioactivities of PACs, effective soil remediation techniques to remove PACs are necessary.

Thermal remediation techniques were previously used to remediate PAC-contaminated soil through processes such as incineration (Acharya and Ives, 1994), thermal desorption (Biache et al., 2008; Renoldi et al., 2003), pyrolysis (Song et al., 2019), and steam injection or steam enhanced extraction (SEE) (Davis, 2003; Kuhlman, 2002; Trine et al., 2019). Recent work on SEE demonstrated both the removal and recovery of PAHs and the formation of OPAHs and hydroxylated-PAHs during SEE through abiotic process (Trine et al., 2019). The increase in OPAH and hydroxylated-PAH concentrations during SEE was linked to an increase in developmental toxicity in the post-SEE soil extracts studied using the zebrafish (*Danio rerio*) developmental toxicity testing (Trine et al., 2019).

While prior work on thermal desorption observed alkyl-PAH and heterocyclic-PAC concentrations decrease post-treatment (Biache et al., 2008), less is known about alkyl-PAHs, azaarenes, PASHs, and OPACs pre- and post-SEE. The concentrations of available PAHs using mild-solvent extraction of soil post-remediation have been previously characterized (Larsson et al., 2013), but the freely dissolved concentrations of other PACs are typically not analyzed and represent an important knowledge gap. The bioactivity of soils pre- and post-SEE are also not known. Bioactivity assessment of soil pre- and post-SEE can provide important additional information on the efficacy of SEE since these assays cover important, potentially toxic effects of all PACs in the samples. To address these research gaps, chemical and bioassay analyses were performed on soil samples pre- and post-SEE. Soil from a Superfund site in Wyckoff/Eagle Harbor, WA, USA (Office of Solid Waste and Emergency Response, 2005; Trine et al., 2019) was characterized with chemical analyses and three *in vitro* reporter gene bioassays pre- and post-SEE.

## 2. Materials and methods

### 2.1. Chemicals and materials

**Fig. 1.**
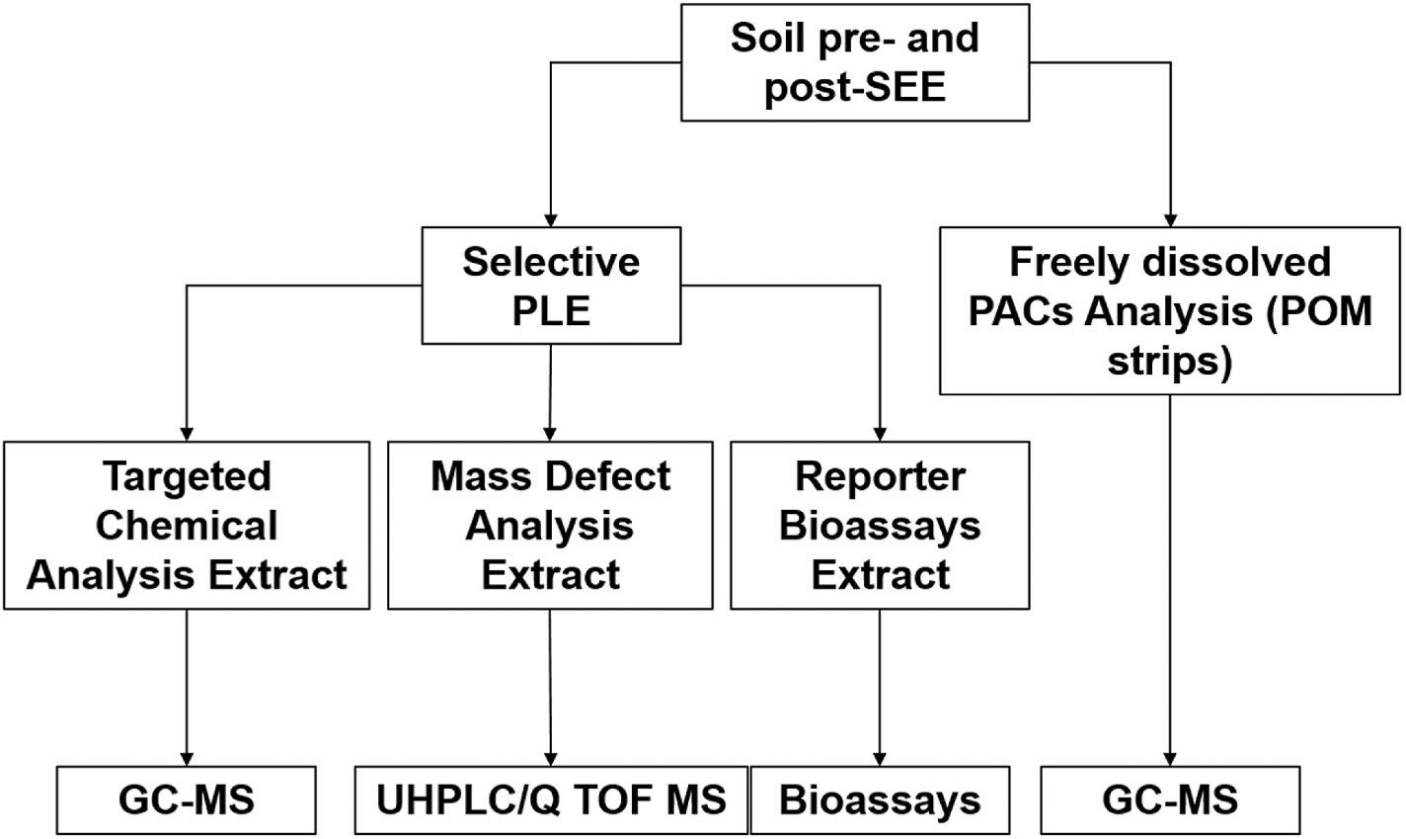
Workflow of all the analyses performed in this study.

The workflow of all the analyses is illustrated in Fig. 1. Individual target PACs, internal and recovery standards (IS and RS, respectively), their abbreviations, predicted log K_ow_, vendors, and purities are listed in the Supplementary Material (SM) (Table S1). Targeted chemical analysis included 87 PACs. Acetone (≥99.8%), *n*-hexane (≥98%), toluene (>99.7%), and anhydrous sodium sulfate (Na_2_SO_4_) (99%) were purchased from VWR (Stockholm, Sweden). Dichloromethane (99.8%) and formic acid (98–100%) were purchased from Sigma Aldrich (Stockholm, Sweden). Methanol (>99%, HPLC grade) and ultrapure water (LC-MS grade) were purchased from Fisher Scientific (Göteborg, Sweden). Calcium chloride and sodium azide were purchased from Merck (Darmstadt, Germany). Strips of polyoxymethylene (POM) (76 μm thickness) were purchased from CS Hyde (Lake Villa, IL, USA).

Dimethyl sulfoxide (DMSO) (99.9%), 17β-estradiol (E2) standard (≥98%), flutamide standard (≥99%) and dihydrotestosterone (DHT) (≥99%) were purchased from Sigma Aldrich (Stockholm, Sweden); 2,3,7,8-tetrachlorodibenzo-*p*-dioxin (TCDD) standard (99.1%) was purchased from AccuStandard (New Haven, CT, USA); and steadylite plus^™^ reporter gene assay system was purchased from PerkinElmer (Hägersten, Sweden).

### 2.2. Soil samples, collection, and laboratory scale SEE

Soil samples analyzed in this study were the same soil as described in the previous study (Trine et al., 2019). The soil was collected in 1995 from a creosote contaminated Wyckoff/Eagle Harbor Superfund site on Bainbridge Island, WA, USA, stored in 4 °C and underwent laboratory-scale SEE remediation in 2015 (Davis, 2003; Trine et al., 2019). Briefly, galvanized carbon steel column (5.1 cm diameter, 15 cm long) was packed with 250 g contaminated soil and was connected to a steam generator and a heat exchanger. The steam generator produced steam at approximate temperature of 130 °C and was injected at the top of the column, which resulted in a downward flow. The soil was marine sand and gravel with 1.2% organic matter content, 12% water content, and 7.7 pH (Trine et al., 2019). Pre- and post-SEE remediated soils were shipped for analysis in this work in 2019 from U.S.A to Sweden and were stored in −20 °C prior to analysis.

### 2.3. Soil extraction

Soil samples pre- and post-remediation were extracted in duplicates using a selective pressurized liquid extraction (SPLE) with basic silica (Titaley et al., 2020a). Approximately 0.5 g of soil was mixed with 2.5 g anhydrous Na_2_SO_4_ (1:5 soil: Na_2_SO_4_). Each 34 mL stainless steel SPLE extraction cell was filled with a cellulose filter in the bottom, 4 g basic silica, and the soil:Na_2_SO_4_ mix. The cell was filled to the top with Na_2_SO_4_ (*i.e.*, within 1 cm from the top). Another cellulose filter was fitted prior to capping of the cell. The extraction was performed using the ASE 350 Accelerated Solvent Extraction system (Thermo Scientific Dionex, Stockholm, Sweden) with dichloromethane as the solvent in two cycles. Extraction blank containing 0.5 g of Na_2_SO_4_ and sand blank (SEE blank) underwent the same extraction procedure as the soil samples. Sample extracts were evaporated to −5 mL using a rotary evaporator (Heidolph, Schwabach, Germany) and to −1 mL under gentle stream of nitrogen gas. The extract was then gravimetrically split for targeted chemical analysis, reporter gene bioassay analysis, and mass defect suspect screening. The extracts for the targeted chemical analysis were spiked with IS mixtures and solvent exchanged to toluene, evaporated to 500 μL, and spiked with RS prior to analysis. Spiking of IS, and subsequently RS, mixtures occurred after the split to enable targeted analysis and bioreporter assays of the same extract. The extract for bioreporter assay analysis was solvent exchanged to DMSO, while the extract for mass defect suspect screening was solvent exchanged to methanol.

### 2.4. Freely dissolved PACs in soil analysis

Assessment of freely dissolved PACs in this study was performed using the POM passive sampler method based on a previous work (Enell et al., 2016), with a few modifications. The POM strips (2 × 4 cm, 0.1 g) were washed in n-hexane for 24 h on a shaker, washed with ultrapure water, dried with tissue, and stored until use at room temperature in an amber glass jar. Approximately 10 g (wet weight) of pre- and post-SEE soils were added to each Teflon-lined amber glass vials, followed by a POM strip. Approximately 35 mL of water with 0.001 M calcium chloride and 0.015 M sodium azide (biocide) was added to each vial. The vials were placed on an end-over-end shaker and shaken for 28 d. Following shaking, the POM strips were removed from the vials, rinsed with ultrapure water, dried with a tissue, and stored at −20 °C until extraction. The strips were extracted twice in a sonication bath with 30 mL acetone:*n*-hexane (1:1, v/v) for 3 h each. Pre- and post-SEE POM strip extract pre-concentration steps followed the same procedures as that of the soil extracts.

The freely dissolved, also often referred as porewater concentration (C_pw_) was determined using the following equation (Arp et al., 2015; Hawthorne et al., 2011; Josefsson et al., 2015):

(eq. 1) $$C_{pw}=\frac{C_{POM}}{K_{POM}}$$

where:

*C_pw_* = freely dissolved (porewater) concentration (ng mL^−1^)

*C_POM_* = POM concentration (ng g^−1^)

*K_POM_* = POM-porewater partitioning coefficient (L kg^−1^ or mL g^−1^)

The *K_POM_* values for PACs in this study (Table S2) were obtained from prior works (Hawthorne et al., 2011; Josefsson et al., 2015) and through a single linear regression model with input of number of carbons for PACs with no assigned log *K_POM_* values (Hawthorne et al., 2011).

### 2.5. Soil and POM strips chemical analysis

An Agilent 7890A gas chromatograph (GC) coupled with a 5975C mass spectrometer (MS) (Santa Clara, CA, USA) was used to analyze and quantify the 87 PACs extracted from the soil and the POM films. The Agilent Select PAH capillary column (30 m × 0.25 mm × 0.15 μm) was used for separation. The extract was injected in splitless mode and the analysis was performed in selected ion monitoring (SIM) mode, with the MS operated in electron ionization (EI) mode. Further details on the GC-MS method are provided elsewhere (Larsson et al., 2018; Titaley et al., 2020a). The recoveries of PACs in prior work using this technique were between 30 and 130% (Titaley et al., 2020a). The PAC concentrations in soil reported in this study were on a dry matter (d.m.) basis.

### 2.6. Reporter gene bioassay analyses

Bioactivity of the soil extracts were measured using three *in vitro* reporter gene bioassays: the aryl hydrocarbon receptor (AhR)-activation using the H4llE-*luc* cell line (DR-CALUX^®^) (Aarts et al., 1995), and the estrogen receptor (ER)-activation (ERα-CALUX^®^) and androgen receptor (AR)-inhibition (anti-AR-CALUX^®^), both of which used the stably transfected human osteoblastic (U2-OS) cell line (Legler et al., 1999; Sonneveld et al., 2005; van der Burg et al., 2010).

#### 2.6.1. DR-CALUX^®^ assay

Four-fold (1:4, v/v) dilution series was prepared in culture medium (α-mem) for every soil extract. In a 96 well plate, triplicates of six concentrations of two extracts and eight concentrations of the TCDD standard (0–300 pM, triplicate wells) were added. Further details on this reporter gene bioassay analysis can be found elsewhere (Aarts et al., 1995; Larsson et al., 2018).

#### 2.6.2. ERα- and anti-AR-CALUX^®^ assays

Steroid-hormone disrupting activities, namely ER agonistic activity and AR antagonistic activity, were measured using the ERα-CALUX^®^ and anti-AR CALUX^®^ assays, respectively, which were derived from the U2-OS cell line. The assays were performed as previously described (Sonneveld et al., 2005; van der Burg et al., 2010), with minor modifications described here. Cultured U2-OS cells were trypsinized and re-suspended in assay medium (DMEM/F12 without phenol red) containing stripped fetal calf serum and seeded into 96-well plates in aliquots of 100 μL and incubated at 37 °C, 5% CO_2_, and high humidity for 24 h. Following incubation, the medium was replaced with assay medium containing standard chemicals or soil extracts for agonistic and antagonistic response testing. Soil extracts were prepared in four-fold (1:4) serial dilutions and tested in six different concentrations in the ERα-CALUX^®^ assay and five concentrations in the anti-AR-CALUX^®^ assay.

For the anti-AR-CALUX^®^ assay, serial dilutions were prepared with medium containing the agonist dihydrotestosterone (DHT) at a concentration of 300 pmol L^−1^. Two sample extracts were tested in each plate and each concentration was added in triplicate wells with a final volume of 100 μL per well and a DMSO concentration of 0.1%. In the ERα-CALUX^®^ assay a standard concentration series of E2 (0–100 pmol L^−1^) was added to each plate in triplicate wells and in the anti-AR-CALUX^®^ assay a standard concentration series of flutamide (0–10 μmol L^−1^) and competing agonist DHT at a constant concentration of 300 pmol L^−1^ was tested on each plate.

#### 2.6.3. Measurement and data analysis of reporter gene bioassay activities

Following a 24 h incubation, the exposure was finished, and cells were washed twice with 100 μL phosphate buffered saline (PBS) containing magnesium (Mg) and calcium (Ca). Approximately 25 μL steadylite plus^™^ substrate and 25 μL PBS with Mg and Ca were added to the cells and plates were stored in darkness at room temperature in 15–20 min. for cell lysis and enzymatic reaction to take place. Cell lysates were then transferred to white 96 well microtiter plates and luciferase activity in each well was measured in a luminometer (FLUOstar^®^ Omega). Concentration-response curves for samples and standards were then analyzed using a sigmoidal concentration-response (variable slope) equation for DR-CALUX^®^ and ERα-CALUX^®^, and a sigmoidal inhibition-response (variable slope) equation for anti-AR-CALUX^®^ (GraphPad Prism^®^ 8.0 software).

Reporter gene bioassays derived TCDD equivalents (bio-TEQs) and estradiol equivalents (bio-EEQs) were calculated based on the concentration-response curves by relating the luciferase induction potencies of the sample extracts to the induction potencies of the standards as described previously (Larsson et al., 2018, 2013). Bio-TEQ concentrations were calculated based on effective concentrations at 25% the maximum induction of TCDD (EC_25_) and bio-EEQs were calculated based on effective concentrations for 10% (EC_10_) of E2 maximum induction. Reporter gene bioassay derived flutamide equivalents (bio--FEQ) was calculated from the concentration-inhibition curves by relating the luciferase inhibition potency of the extract to the luciferase inhibition potency of the standard and based on concentrations for 25% of maximal inhibition of flutamide (IC_25_). The chemically derived TCDD equivalents (chem-TEQs) (Table S1) were calculated based on the multiplication of the concentration of the compound in the extract and the assigned relative effect potency (REP) value of the compound (Lam et al., 2018a; Larsson et al., 2014, 2012), as previously described (Larsson et al., 2013).

### 2.7. Mass defect suspect screening analysis

Suspect screening was performed using ultra high pressure liquid chromatography coupled with quadrupole time-of-flight MS (UHPLC/Q-TOF MS). The UHPLC/Q-TOF MS analysis was performed using an Agilent 1290 Infinity II LC system coupled with a 6545 Q-TOF MS with a dual jet stream electrospray ion source (Santa Clara, CA, USA). The analysis was performed based on a previous work (Tian et al., 2017), with a few modifications. The Waters ACQUITY UPLC BEH C18 column (2.1 mm × 100 mm, particle size 1.7 μm) was used for separation. The mobile phase consisted of 0.1% (v/v) formic acid in water (A) and methanol (B), delivered at a flow rate of 0.2 mL min^−1^. The Q-TOF MS was performed in full scan mode and operated in electrospray ionization (ESI) + mode, scanning for *m/z* 100–1000 with a resolution power >15,000.

Mass defect analysis was performed to determine the diversity of azaarenes and OPACs post-SEE due to their known toxicities and bioactivities (Bleeker et al., 2002; Hinger et al., 2011; Lundstedt et al., 2007; Machala et al., 2001). The overall workflow for mass defect suspect screening is shown in the SM (Fig. S1). The UHPLC/Q-TOF MS raw data were centroided and converted to the mzML format with msConvert (Chambers et al., 2012) and pre-processed with PatRoon package in the R programming language (Helmus et al., 2021). Feature detection was conducted using the OpenMS algorithm (Röst et al., 2016), resulting in a total of 5165 features across extracts pre- and post-SEE. Peak grouping, also performed with OpenMS, was applied with intensity filtering above 30,000 and higher than three times peak area to blank samples, resulting in 1056 peak groups. Finally, mass defect plots was performed with an in-house script developed in R (R Core Team, 2020). The isomers tentatively identified in the analysis were divided to three categories: decreased, no change, and increased, based on their area counts comparison pre- and post-SEE. Isomers with decreased response meant that the area count of the isomer decreased more than double post-SEE; isomers with no change meant that the area count of the isomer decrease or increase post-SEE, but the magnitude was less than double; and isomers with increased response meant that the area count of the isomer increased more than double post-SEE.

### 2.8. Quality assurance/quality control (QA/QC)

Quantification of PACs were performed by GC-MS using the isotope dilution method. Relative standard deviation of the relative response factors was ≤20% for all PACs, except for OPAHs where the relative standard deviation was <28%. Limit of Quantitation (LOQ) was calculated by multiplying the concentration of the analytes in the SEE blank 10× or based on the lowest point of the calibration curve if no peak was detected in the blank (Tables S3 and S4). Non-detects were substituted with zero for the concentration summation.

In the reporter gene bioassay analyses, only plates with ≥15% standard deviation within triplicates, EC_50_ values of TCDD and estradiol from 8 to 18 pmol L^−1^ and from 1.9 to 19 pmol L^−1^, respectively, and an IC_50_ value of flutamide from 1.0 to 10 μmol L^−1^ were used in calculations of bioassay derived equivalents (bio-TEQs, bio-EEQs or bio-FEQs). Mean luciferase activity of DMSO control triplicates + three times standard deviation were used to determine limit of detection (LOD) in all three assays (Larsson et al., 2018).

Mass defect suspect screening tentatively identified additional PACs at identification level 4 (Schymanski et al., 2014), by confirming the [M+H]^+^ peak isotope masses and abundances.

## 3. Results and discussion

### 3.1. Profile of PACs in soil pre- and post-SEE

**Fig. 2.**
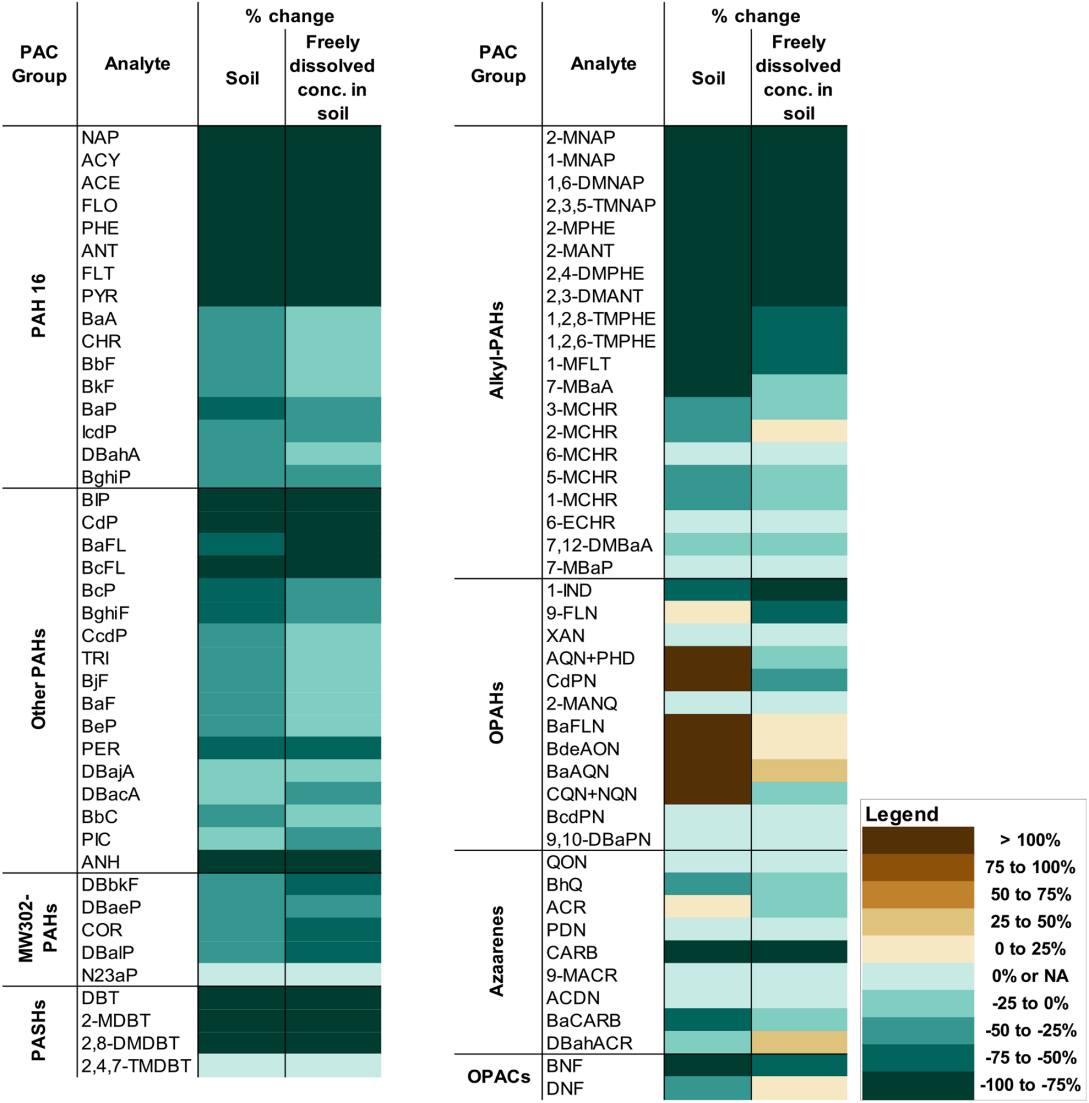
Change in concentrations of PAC-groups in soil and freely dissolved concentrations of PAC-groups in soil (%) pre- and post-SEE (*n* = 2). NA indicated < LOQ. Color symbols: https://ColorBrewer2.org.

Summed mean PAC concentrations (∑_PACs_) in the soil decreased post-SEE (940 μg g^−1^ to 71 μg g^−1^), but ∑_OPAHs_ increased post-SEE (8.4 μg g^−1^ to 24 μg g^−1^), and summed mean PAH 16 concentrations (∑_PAH 16_) decreased by 92% post-SEE (Table S5). The percent removal of several two-to four-rings PAH 16, namely naphthalene, acenaphthylene, acenaphthene (ACE), fluorene (FLO), phenanthrene (PHE), anthracene (ANT), fluoranthene (FLT), and pyrene (PYR), in the soil (88–100%) were higher than the percent removal of the other four and higher number of ring PAHs in soil (27–51%) (Fig. 2). The sum of the other 22 PAHs not included in PAH 16 (∑_Other parent-PAHs_), decreased by 72% post-SEE (Fig. 2). Similar difference in the percent removal between lower and higher number of rings PAHs recorded for PAH 16 were also observed for the 22 PAHs and indicated the persistence of larger PAHs, which posed potential environmental health problem.

The removal of MW302-PAHs was the lowest (27–35%) compared to other PACs (Fig. 2), which was likely due to the strong sorption of MW302-PAHs to soil (Chiou et al., 1998). The concentrations of MW302-PAHs (< LOQ to 0.23 μg g^−1^) were also the lowest of all other groups of compounds pre-SEE (Table S5). The MW302-PAHs results in this study were in agreement with the observation in the previous SEE study (Trine et al., 2019).

The concentrations of alkyl-PAHs (< LOQ to 21 μg g^−1^) decreased post-SEE (1–100%) (Fig. 2). Comparable magnitude of removals between FLT, PHE, ANT, chrysene (CHR), and their respective alkyl-PAH analogues were observed (Fig. 2). The removal of alkylated CHRs were lower, and 7,12-dimethylbenzo [*a*]anthracene was the lowest, compared to the rest of alkyl-PAHs (Fig. 2). The alkyl-PAHs results in this study demonstrated the ability of SEE to remove these compounds in addition to PAHs, likely through the same extraction or thermal abiotic degradation process as that of parent-PAHs (Andersson et al., 2003; Davis, 2003; Trine et al., 2019).

The only group of compounds studied that had increased in concentrations post-SEE were the OPAHs (Fig. 2), which was in agreement with a previous study findings (Trine et al., 2019). The increase in OPAH concentrations post-SEE were partially attributed to the degradation of PAHs and subsequent formation of OPAHs during SEE (Trine et al., 2019). Introduction of steam in SEE could contribute to OPAH formation (Andersson et al., 2003). The concentration of FLO and benzo [*a*]fluorene decreased by 100 and 68% post-SEE, respectively, while the concentration of 9-fluorenone (9-FLN) and benzo [*a*]fluorenone (BaFLN) increased by 11 and 190%, respectively (Fig. 2). Similarly, the concentration of benzo [*a*]anthracene went down 47% post-SEE, while the concentration of benzo [*a*]anthracene-7,12-dione (BaAQN) went up 140% (Fig. 2). Both PHE and ANT were removed ≥98% and the concentrations of anthracene-9,10-dione + phenanthrene-1,4-dione (AQN + PHD) increased by 140%, indicating that degradation of other PAHs beyond the parent compounds of OPAHs included in the targeted chemical analysis in this study contributed to the increase in OPAH concentrations post-SEE.

The summed mean concentrations of azaarenes (∑_azaarenes_, 18 μg g^−1^), PASHs (∑_PASHS_, *7.2* μg g^−1^), and OPACs (∑_OPACs_, 4.5 μg g^−1^) decreased by 85, 98, and 84%, respectively, post-SEE (Fig. 2). Carbazole (CARB) was the azaarene with the greatest removal during SEE (98%), but the concentration of acridine (ACR) remained unchanged (Fig. 2). The difference in removal between these compounds was hypothesized to be due to the higher stability of ACR, a tertiary amine, relative to CARB, a secondary amine (Schwarzenbach et al., 2005). All of the measured PASHs were removed during SEE (98–100%), with 2, 8-dimethyldibenzothiophene removed to < LOQ level (Fig. 2). Of the two OPACs studied, benzo [*b*]naphtho [2,1-*d*]furan was more removed (85%) than dinaphtho [2,1-*b*:1′,2’-]furan (DNF) (26%). The presence of an extra ring in DNF, which increased the stability of the compound, likely reduced the removal rate of the compound. The heterocyclic-PAC results indicated the ability of SEE to remove heterocyclic compounds, in addition to PAHs and alkyl-PAHs.

### 3.2. Freely dissolved PAC concentrations in soil pre- and post-SEE

Freely dissolved concentrations (C_pw_) of PACs were calculated using eq. (1). The freely dissolved concentrations of PACs in soil were summed and were found to decrease post-SEE (−88%) (Fig. 2). Concentration decreases (91–100%) for freely dissolved PAH 16 with two-to four-rings were similar to concentration decreases in soil (Fig. 2), indicating that SEE also impacted the freely dissolved PAH 16. Similar trend was also observed for two-to four-ring other PAHs. Freely dissolved concentrations of PAHs with four-ring or more were lower compared to compounds with less than four rings, likely because these compounds remained bound to soil (Chiou et al., 1998; Smulek et al., 2020).

The decrease in freely dissolved MW302-PAHs (46–60%) was higher than the removal of MW302-PAHs in soil (27–35%) (Fig. 2), but the freely dissolved concentrations of MW302-PAHs were low pre- and post-SEE (0.00016 and 0.000073 ng mL^−1^, respectively).

Alkyl-PAHs with lower number of rings had higher percent removal than alkyl-PAHs with higher number of rings (Table S5). The decrease in the freely dissolved concentrations of five alkyl-PAHs (7-methylbenzo [*a*]anthracene, 1-, 2-, 3-, and 5-methylchrysene (MCHR)) post-SEE (0–22%) was lower than the reduction of other alkyl-PAHs in soil (Fig. 2). However, the freely dissolved concentrations of these analytes in soil were low post-SEE (0.0061–0.023 ng mL^−1^), therefore the risk from these compounds appeared to be limited.

The freely dissolved concentrations of five of the eight OPAHs decreased post-SEE (6–85%) (Fig. 2), indicating that some of the OPAHs formed during SEE in the soil were not available. However, the concentrations of freely dissolved BaFLN, 7H-benzo [*de*]anthracen-7-one, and BaAQN increased in the post-SEE soil by 7, 23, and 29%, respectively, but the levels post-SEE were low (0.029–0.46 ng mL^−1^).

The removal of freely dissolved heterocyclic-PACs varied depending on the substitutions in the ring. For azaarenes, the decrease in freely dissolved concentrations were less than the removal in soil (Table S5). Freely dissolved PASHs were removed in the same percent as that of PASHs in soil. Of the two OPACs studied, the decrease in freely dissolved concentration of benzo [*b*]naphtho [2,1-*d*]furan was comparable to the removal in soil, but there was a slight increase in freely dissolved concentration of dinaphtho [2,1-*b*:1′,2’-]furan (DNF) post-SEE (11%) (Fig. 2). The higher freely dissolved concentrations of heterocyclic-PACs pre- and post-SEE, with concentrations up to 53 ng mL^−1^ pre-SEE, were attributed to the polarity of these compound, which suggested a potential risk in non-remediated, creosote-contaminated soil.

### 3.3. Reporter gene bioassay analysis of soil pre- and post-SEE

The bioactivities of the post-SEE soil decreased for all reporter bioassays (Table 1). The bio-TEQ concentrations decreased pre- and post-SEE (73–27 ng g^−1^) and the bio-TEQ levels pre-SEE were similar to the bio-TEQ concentrations previously measured in PAH- or creosote-contaminated soils collected from various sites in Sweden, including from an abandoned gasworks plant, a railway station, a former wood preservation facility, and a steel industrial area (Larsson et al., 2018, 2013). The ER activation (bio-EEQ) and AR inhibition (bio-FEQ) concentrations decreased pre- and post-SEE (1.9–0.60 ng g^−1^ and 1,100,000 to 84,000 ng g^−1^, respectively). The magnitudes of bio-EEQ and -FEQ pre-SEE were comparable to the magnitude of activities measured in different contaminated soil extracts collected from a pesticide dumping site in Tajikistan (Pieterse et al., 2015). Prior studies that used ERα-CALUX^®^ and other assays with the same mode of activity observed endocrine disruption by PACs such as PAHs (Boonen et al., 2020), alkyl-PAHs (Lee et al., 2017), PASHs (Brinkmann et al., 2014), and OPACs (Brinkmann et al., 2014; Vondráček et al., 2004). Therefore, the post-SEE decrease in bioactivity measured in the ERα-CALUX^®^ bioassay in this study was likely attributed to the decreased concentrations of these compounds in soil post-SEE.

The change in the concentration of known AhR-active compounds could be measured using chem-TEQs. The chem-TEQs of OPAHs and azaarenes increased post-SEE, while the chem-TEQs of the rest of PACs decreased (Table S6). The greatest chem-TEQ decrease was measured for PASHs, followed by OPACs. Of the compounds with assigned REP-values, benzo [*k*]-, [*b*]-, and [*j*]-fluoranthenes were the three compounds with the highest chem-TEQs pre- and post-SEE (Table S6). High chem-TEQ contributions were also measured for CHR and its alkylated isomers, namely 2- and 3-MCHR (Table S6). The chem-TEQs of all OPAHs, with the exception of BaFLN, 7H-benzo [*de*]anthracen-7-one, BaAQN, CQN, and NQN, were not available due to the lack of REP data or because the compounds were <LOQ in the soil. The chem-TEQ contributions of the heterocyclic-PACs were limited.

Pre-SEE chem-TEQs were only able to explain 26% of the overall bio-TEQ (19 and 73 ng g^−1^ d.m., respectively), while post-SEE chem-TEQs were able to explain up to 44% of the overall bio-TEQ (12 and 27 ng g^−1^ d.m., respectively). The gap between chem- and bio-TEQs indicated the presence of AhR-active compounds beyond the list of compounds in this study, but these unknown AhR-active compounds were likely removed as indicated by the increase in chem-TEQ contribution toward bio-TEQ post-SEE.

Compounds such as BcFL, MW302-PAHs, and 5-MCHR that were known to be toxic and bioactive (Richter-Brockmann and Achten, 2018; Vondráček et al., 2020), but their REP values were yet to be calculated and, thus, their bioactivities were undetermined. The risk of contaminated soil is also related to the availability of the compounds post-SEE, and it is possible that bioassay analysis of the bioavailable fraction measured by the POM strips had shown an increase in bioactivity post-SEE, even though the bioactivity in the soil decreases (Andersson et al., 2009). Therefore, the combination of chemical and *in vitro* or genotoxicity tests could provide a complete picture on the efficacy of soil remediation studies (Titaley et al., 2020b).

### 3.4. Mass defect suspect screening of azaarenes and OPAC pre- and post-SEE

The UHPLC/Q-TOF MS mass defect suspect screening analysis revealed the presence of 27 tentatively identified unique isomers of azaarenes and OPAC (Table S7) within a mass error <5 ppm of theoretical masses. The profile of the tentatively identified azaarenes in this study were largely similar to the profile of isomers identified in a previous study (Tian et al., 2017). Tian et al. did not identify any benzo-carbazole isomers, but 15 isomers of this compound were detected in this study (Table S7). Only one OPAC, benzonaphthofuran, was measured in soil and three isomers of this compound were tentatively identified in the soil. The tentative identification of these isomers further illustrated the presence of other compounds that could contribute to the bioactivities in the soil extracts.

Comparison of pre- and post-SEE soil extracts showed five tentatively identified PAC isomers with increased responses post-SEE, which consisted of one phenanthridine isomer, two methyl-benzoacridine isomers, one azabenzopyrene isomer, and one dimethyl-dibenzoacridine isomer (Table S7). Of these isomers, phenanthridine and ACR were measured in the targeted chemical analysis, both of which were removed post-SEE (Table S5). There are currently no known metabolic pathways that would lead to the formation of azaarenes and prior bioremediation study involving azaarenes was not able to explain the increase in azaarenes concentration (Lemieux et al., 2009).

Beyond the tentatively identified isomers that increased post-SEE, 24 of 27 azaarenes had at least one isomer with no change in response post-SEE, which indicated the potential persistence of some azaarenes post-remediation (Table S7). Based on a previous alkyl-PAHs study, the bioactivities of alkylated-compounds were higher than the parent compounds (Lam et al., 2018a). Therefore, the bioactivities of alkylated azaarenes are likely to be different than azaarenes, with the alkylated azaarenes potentially exhibiting greater bioactivity than azaarenes.

## 4. Conclusions

The combination of chemical and bioassay analyses indicated the ability of SEE to remove and reduce alkyl-PAHs and heterocyclic-PACs in soil. The freely dissolved alkyl-PAHs and heterocyclic-PACs in soil were reduced post-SEE. The overall removal was greater for PACs with lower number of rings and lower for those with higher number of rings, but the availability was generally greater for lower number rings and more polar compounds like OPAHs and heterocyclic-PACs. Three *in vitro* reporter gene bioassays measured decreases in bioactivities post-SEE. Mass defect suspect screening measured increased responses of five azaarenes post-SEE. A limitation of this study is the lack of statistical analysis due to duplications of the samples. The comprehensive analyses in this study included a list of compounds beyond PAH 16. However, unless the list of PAHs or PACs to be regulated is expanded by the regulatory agencies, only PAH 16 results are considered relevant. Removal of PACs and formation of their degradation products during lab-scale SEE study occurred in an extremely short amount of time, in the order of hours, yet field-scale application of SEE remediation occurs over months to years, and the results of this short term laboratory experiment does not provide information on the long term fate of these compounds in the subsurface after SEE. Additional samples and comparison to other thermal techniques are recommended as well as chemical analysis of the soil samples from field sites before and after thermal remediation. Non-targeted analysis can provide additional insights into the presence of non-PACs compound in the soil samples after a field-scale application of SEE or another thermal technology. Additional reporter gene bioassays with other modes of action, like measurement of genotoxicity, mutagenicity, or oxidative stress, and in combination with bioassay analysis of freely dissolved PACs in soils, would give a more comprehensive picture of the risks of PACs and their polar transformation products post-SEE.

## Supplementary Material

1

## Figures and Tables

**Table 1.** Mean bio-TEQ concentrations (ng g^−1^ or μg g^−1^ d.m.) of soil extracts pre- and post-SEE (*n* = 2). Bioactivities are expressed in equivalences of the standard reference compound in each respective assay. No responses were measured in the SEE blank.

Soil extract	DR-CALUX^®^	ERα-CALUX^®^	Anti-AR-CALUX^®^
	(AhR activity)	(ER activity)	(AR antagonistic activity)
		
	Bio-TEQ (EC_25_) ng g^−1^ d.m.	EEQ (EC_10_) ng g^−1^ d.m.	FEQ (IC_25_) ng g^−1^ d. m
Pre-SEE	73	1.9	1,100,000
Post-SEE	27 (−63%)	0.60 (−68%)	<LOQ to 84,000 (−91%)

## Data Availability

Data will be made available on request.
