# Supplementary material for: Extensive chemical and bioassay analysis of polycyclic aromatic compounds in a creosote-contaminated superfund soil following steam enhanced extraction
Source: Environ Pollut. Author manuscript; Available in PMC 2023 Jan 23. (PMC9869926; doi:10.1016/j.envpol.2022.120014)
Supplement: 1 [file NIHMS1863152-supplement-1.docx]

**Supplementary Material**

**Extensive Chemical and Bioassay Analysis of Polycyclic Aromatic Compounds**

**in a Creosote-Contaminated Superfund Soil following Steam Enhanced Extraction**

Ivan A. Titaley^a,1,*^, Lisandra Santiago Delgado Trine^b,2^, Thanh Wang^a^, Daniel Duberg^a^, Eva L. Davis^c^, Magnus Engwall^a^, Staci L. Massey Simonich^b,d^, Maria Larsson^a^

^a^Man-Technology-Environment (MTM) Research Centre, School of Science and Technology, Örebro University, Örebro SE-701 82, Sweden

^b^Department of Environmental and Molecular Toxicology, Oregon State University, Corvallis, Oregon 97331, USA

^c^Center for Environmental Solutions & Emergency Response, Groundwater, Watershed and Ecosystems Restoration Division, United States Environmental Protection Agency, Ada, Oklahoma 74820, USA

^d^Department of Chemistry, Oregon State University, Corvallis, Oregon 97331, USA

^*^Corresponding Author: ivan.titaley@oregonstate.edu

Phone: +1 541 737 9208, Fax: +1 541 737 0497

^1^Present address: Department of Environmental and Molecular Toxicology, Oregon State University, Corvallis, Oregon 97331, USA

^2^Present address: Center for Public Health and Environmental Assessment, Pacific Ecological Systems Division, Ecology Effects Branch, Integrated Stable Isotope Research Facility, United States Environmental Protection Agency, Corvallis, Oregon 97333, USA

**Supplementary Material contains:**

20 pages

1 figures

7 tables

**Content:**

**Table S1.** List of target PACs, internal standards (IS), and recovery standard (RS) used in this study and their abbreviations, vendors, purities, quantifier ions, and assigned relative effect potency (REP) values 3

**Figure S1.** Mass defect suspect screening analysis workflow 8

**Table S2.** List of log *K_POM_* for all PACs. 9

**Table S3.** LOQs of PACs in soil. 10

**Table S4.** LOQs of PACs in POM films 11

**Table S5.** PAC concentration in soil and in POM films, and freely dissolved concentrations 12

**Table S6.** Chem-TEQs of PACs with assigned REP values in the soil extracts based on the H4IIE-*luc* reporter bioassay 16

**Table S7.** Isomers of azaarenes and OPACs detected using the mass defect suspect screening approach. 18

**References** 20

**Table S1.** List of target PACs, internal standards (IS), and recovery standard (RS) used in this study and their abbreviations, vendors, purities, quantifier ions, assigned relative effect potency (REP) values.

| **Compound** | **Abbreviation** | **Purity (%)** | **Vendor** | **Log K_ow_^a^** | **IS** | **REP** |
| --- | --- | --- | --- | --- | --- | --- |
| ***PAH 16*** |  |  |  |  |  |  |
| Naphthalene | NAP | NA^b^ | NIST^c^ | 3.3 | *d_8_-NAP* | 0 |
| Acenaphthylene | ACY | NA^b^ | NIST^c^ | 3.9 | *d_8_-ACY* | 0 |
| Acenaphthene | ACE | NA^b^ | NIST^c^ | 3.9 | *d_10_-ACE* | 0 |
| Fluorene | FLO | NA^b^ | NIST^c^ | 4.2 | *d_10_-FLO* | 0 |
| Phenanthrene | PHE | NA^b^ | NIST^c^ | 4.5 | *d_10_-PHE* | 0 |
| Anthracene | ANT | NA^b^ | NIST^c^ | 4.5 | *d_10_-ANT* | 0 |
| Fluoranthene | FLT | NA^b^ | NIST^c^ | 5.2 | *d_10_-FLT* | 0 |
| Pyrene | PYR | NA^b^ | NIST^c^ | 4.9 | *d_10_-PYR* | 4.97E-06 |
| Benzo[*a*]anthracene | BaA | NA^b^ | NIST^c^ | 5.6 | *d_12_-BaA* | 1.29E-05 |
| Chrysene | CHR | NA^b^ | NIST^c^ | 5.8 | *d_12_-CHR* | 3.95E-05 |
| Benzo[*b*]fluoranthene | BbF | NA^b^ | NIST^c^ | 5.8 | *d_12_-BbF* | 5.37E-04 |
| Benzo[*k*]fluoranthene | BkF | NA^b^ | NIST^c^ | 6.1 | *d_12_-BkF* | 2.32E-03 |
| Benzo[*a*]pyrene | BaP | NA^b^ | NIST^c^ | 6.1 | *d_12_-BaP* | 5.39E-05 |
| Indeno[1,2,3-*cd*]pyrene | IcdP | NA^b^ | NIST^c^ | 6.8 | *d_12_-IcdP* | 4.06E-04 |
| Dibenzo[*a*,*h*]anthracene | DBahA | NA^b^ | NIST^c^ | 6.7 | *d_14_-DBahA* | 1.45E-03 |
| Benzo[*ghi*]perylene | BghiP | NA^b^ | NIST^c^ | 6.6 | *d_12_-BghiP* | 2.62E-06 |
| ***Other parent-PAHs (non PAH 16)*** |  |  |  |  |  |  |
| Biphenyl | BIP | NA^b^ | NIST^c^ | 4.0 | *d_8_-NAP* | NA^b^ |
| 4H-cyclopenta[*def*]phenanthrene | CdP | NA^b^ | NIST^c^ | 4.9 | *d_10_-FLT* | 2.90E-07 |
| Benzo[*a*]fluorene | BaFL | 98 | Ultra^d^ | 5.5 | *d_10_-PYR* | 1.50E-05 |
| Benzo[*c*]fluorene | BcFL | 99.3 | LGC^e^ | 5.4 | *d_10_-PYR* | NA^b^ |
| Benzo[*c*]phenanthrene | BcP | NA^b^ | Chiron Mix^f^ | 5.7 | *d_12_-BaA* | NA^b^ |
| Benzo[*ghi*]fluoranthene | BghiF | NA^b^ | NIST^c^ | 5.7 | *d_12_-BaA* | NA^b^ |
| Cyclopenta[*cd*]pyrene | CcdP | NA^b^ | NIST^c^ | 5.7 | *d_12_-BaA* | NA |
| Triphenylene | TRI | NA^b^ | NIST^c^ | 5.5 | *d_12_-CHR* | 2.90E-07 |
| Benzo[*j*]fluoranthene | BjF | NA^b^ | NIST^c^ | 6.3 | *d_12_-BkF* | 4.16E-04 |
| Benzo[*a*]fluoranthene | BaF | NA^b^ | NIST^c^ | 5.4 | *d_12_-BkF* | NA^b^ |
| Benzo[*e*]pyrene | BeP | NA^b^ | NIST^c^ | 6.4 | *d_12_-BaP* | 7.05E-07 |
| **Compound** | **Abbreviation** | **Purity (%)** | **Vendor** | **Log K_ow_^a^** | **IS** | **REP** |
| Perylene | PER | NA^b^ | NIST^c^ | 6.0 | *d_12_-BaP* | 0 |
| Dibenzo[*a*,*j*]anthracene | DBajA | NA^b^ | NIST^c^ | 6.6 | *d_14_-DBahA* | 5.82E-04 |
| Dibenzo[*a*,*c*]anthracene | DBacA | NA^b^ | NIST^c^ | 6.4 | *d_14_-DBahA* | 5.75E-04 |
| Benzo[*b*]chrysene | BbC | NA^b^ | NIST^c^ | 7.0 | *d_12_-IcdP* | NA^b^ |
| Picene | PIC | NA^b^ | NIST^c^ | 7.1 | *d_14_-DBahA* | NA^b^ |
| Anthanthrene | ANH | NA^b^ | NIST^c^ | 7.0 | *d_12_-IcdP* | NA^b^ |
| ***MW-302 PAHs*** |  |  |  |  |  |  |
| Dibenzo[*b*,*k*]fluoranthene | DBbkF | NA^b^ | NIST^c^ | 7.4 | *d_12_-IcdP* | NA^b^ |
| Dibenzo[*a*,*e*]pyrene | DBaeP | NA^b^ | NIST^c^ | 7.0 | *d_12_-IcdP* | NA^b^ |
| Coronene | COR | NA^b^ | NIST^c^ | 7.6 | *d_12_-IcdP* | NA^b^ |
| Dibenzo[*a,l*]pyrene | DBalP | 99.3 | LGC^e^ | 7.7 | *d_12_-IcdP* | NA^b^ |
| Naphtho[2,3-*a*]pyrene | N23aP | 99 | Ultra^d^ | 7.3 | *d_12_-IcdP* | 1.82E-04 |
| ***Alkyl-PAHs*** |  |  |  |  |  |  |
| 2-methylnaphthalene | 2-MNAP | NA^b^ | Chiron Mix^f^ | 3.9 | *d_10_-1-MNAP* | NA^b^ |
| 1-methylnaphthalene | 1-MNAP | NA^b^ | Chiron Mix^f^ | 3.9 | *d_10_-1-MNAP* | NA^b^ |
| 1,6-dimethylnaphthalene | 1,6-DMNAP | NA^b^ | Chiron Mix^f^ | 3.9 | *d_10_-1-MNAP* | NA^b^ |
| 2,3,5-trimethylnaphthalene | 2,3,5-TMNAP | NA^b^ | Chiron Mix^f^ | 4.7 | *d_10_-1-MNAP* | NA^b^ |
| 2-methylphenanthrene | 2-MPHE | >99 | Chiron Mix^f^ | 4.9 | *d_12_-9-MANT* | 1.50E-07 |
| 2-methylanthracene | 2-MANT | 97 | Sigma^g^ | 5.0 | *d_12_-9-MANT* | 3.20E-07 |
| 2,4-dimethylphenanthrene | 2,4-DMPHE | >99.5 | Chiron Mix^f^ | 5.3 | *d_12_-9-MANT* | 1.30E-07 |
| 2,3-dimethylanthracene | 2,3-DMANT | 99.8 | Chiron Mix^f^ | 5.2 | *d_12_-9-MANT* | 1.10E-06 |
| 1,2,8-trimethylphenanthrene | 1,2,8-TMPHE | 98.5 | Chiron Mix^f^ | 5.3 | *d_12_-9-MANT* | 1.00E-06 |
| 1,2,6-trimethylphenanthrene | 1,2,6-TMPHE | >99.5 | Chiron Mix^f^ | 5.3 | *d_12_-9-MANT* | 9.40E-07 |
| 1-methylfluoranthene | 1-MFLT | NA^b^ | Chiron Mix^f^ | 5.5 | *d_12_-9-MANT* | 1.70E-07 |
| 7-methylbenzo[*a*]anthracene | 7-MBaA | 98 | Sigma^g^ | 5.9 | *Md_3_-5MCHR* | 2.70E-04 |
| 3-methylchrysene | 3-MCHR | 99.3 | Sigma^g^ | 5.7 | *Md_3_-5MCHR* | 2.00E-03 |
| 2-methylchrysene | 2-MCHR | 99.3 | Sigma^g^ | 5.7 | *Md_3_-5MCHR* | 1.20E-03 |
| 6-methylchrysene | 6-MCHR | 98.8 | LGC^e^ | 5.7 | *Md_3_-5MCHR* | NA^b^ |
| 5-methylchrysene | 5-MCHR | 99 | LGC^e^ | 5.7 | *Md_3_-5MCHR* | NA^b^ |
| 1-methylchrysene | 1-MCHR | 99.1 | Sigma^g^ | 5.9 | *Md_3_-5MCHR* | 2.50E-04 |
| 6-ethylchrysene | 6-ECHR | 99.3 | Chiron Mix^f^ | 6.0 | *Md_3_-5MCHR* | 5.00E-06 |
| 7,12-dimethylbenzo[*a*]anthracene | 7,12-DMBaA | NA^b^ | Sigma^g^ | 5.8 | *Md_3_-5MCHR* | NA^b^ |
| **Compound** | **Abbreviation** | **Purity (%)** | **Vendor** | **Log K_ow_^a^** | **IS** | **REP** |
| 7-methylbenzo[*a*]pyrene | 7-MBaP | 98 | Sigma^g^ | 5.8 | *Md_3_-5MCHR* | 1.20E-03 |
| ***OPAHs*** |  |  |  |  |  |  |
| 1-indanone | 1-IND | >99 | Alfa^h^ | 1.9 | *d_8_-9-FLN* | 0 |
| 9-fluorenone | 9-FLN | 98 | Sigma^g^ | 3.6 | *d_8_-9-FLN* | 0 |
| Xanthone | XAN | 99 | Fisher^i^ | 3.4 | *d_8_-9-FLN* | NA^b^ |
| Anthracene-9,10-dione+ Phenanthrene-1,4-dione | AQN+PHD | 99.8+99.5 | Sigma^g^ | 3.4; 3.2 | *d_8_-AQN* | 0 |
| 4H-cyclopenta[*def*]phenanthrenone | CdPN | 99.6 | Sigma^g^ | 3.9 | *d_8_-9-FLN* | 0 |
| 2-methyl-9,10-anthraquionone | 2-MANQ | 97 | Alfa^h^ | 3.3 | *d_8_-9-FLN* | 5.80E-07 |
| Benzo[*a*]fluorenone | BaFLN | 99.8 | Sigma^g^ | 4.7 | *d_8_-9-FLN* | 1.70E-06 |
| 7H-benzo[*de*]anthracen-7-one | BdeAON | 99 | Alfa^h^ | 4.8 | *d_8_-AQN* | 6.20E-07 |
| Benzo[*a*]anthracene-7,12-dione | BaAQN | >98 | Alfa^h^ | 4.4 | *d_8_-AQN* | 1.50E-06 |
| 1,4-chrysenequinone+ Naphthacene-5,12-dione | CQN+NQN | 93+97 | TCI^j^+ Sigma^g^ | 4.0; 4.3 | *d_8_-AQN* | 4.00E-06 |
| 6H-benzo[*cd*]pyren-6-one | BcdPN | 98.8 | Sigma^g^ | 4.3 | *d_8_-AQN* | 1.70E-07 |
| 9,10-dihydrobenzo[*a*]pyren-7(8H)-one | 9,10-DBaPN | 97 | Sigma^g^ | 5.0 | *d_8_-AQN* | 8.70E-07 |
| ***Azaarenes*** |  |  |  |  |  |  |
| Quinoline | QON | 98 | Sigma^g^ | 2.0 | *d_9_-ACR* | 0 |
| Benzo[*h*]quinoline | BhQ | 98 | Alfa^h^ | 3.4 | *d_9_-ACR* | 0 |
| Acridine | ACR | >98 | Alfa^h^ | 3.4 | *d_9_-ACR* | 1.60E-07 |
| Phenanthridine | PDN | 98 | Fisher^i^ | 3.5 | *d_9_-ACR* | NA |
| Carbazole | CARB | 99.3 | Sigma^g^ | 3.6 | *d_8_-CARB* | 0 |
| 9-methylacridine | 9-MACR | 99 | Chiron^k^ | 3.2 | *d_9_-ACR* | 0 |
| Acridone | ACDN | 99 | Sigma^g^ | 2.2 | *d_9_-ACR* | 0 |
| 11H-benzo[*a*]carbazole | BaCARB | 99.8 | Chiron^k^ | 4.8 | *d_8_-CARB* | 0 |
| Dibenzo[*a*,*h*]acridine | DBahACR | 99.6 | LGC^e^ | 5.7 | *d_9_-ACR* | 3.60E-03 |
| ***PASHs*** |  |  |  |  |  |  |
| Dibenzothiophene | DBT | 98.7 | Chiron Mix^f^ | 4.4 | *d_8_-DBT* | 4.60E-08 |
| 2-methyldibenzothiophene | 2-MDBT | >96.5 | Chiron Mix^f^ | 4.9 | *d_8_-DBT* | 5.20E-08 |
| 2,8-dimethyldibenzothiophene | 2,8-DMDBT | >96.5 | Chiron Mix^f^ | 4.9 | *d_8_-DBT* | 7.60E-07 |
| 2,4,7-trimethyldibenzothiophene | 2,4,7-TMDBT | NA^b^ | Chiron Mix^f^ | 5.0 | *d_8_-DBT* | NA^b^ |
| **Compound** | **Abbreviation** | **Purity (%)** | **Vendor** | **Log K_ow_^a^** | **IS** | **REP** |
| ***OPACs*** |  |  |  |  |  |  |
| Benzo[*b*]naphtho[2,1-*d*]furan | BNF | 98.8 | Sigma^g^ | 5.0 | *d_8_-9-FLN* | 6.90E-07 |
| Dinaphtho[2,1-*b*:1',2'-]furan | DNF | >96 | Chiron^k^ | 6.0 | *d_8_-AQN* | NA^b^ |
| ***PAHs IS*** |  |  |  |  |  |  |
| Naphthalene-*d8* | *d_8_*-NAP | 97.5 | Labor^l^ | NA^b^ | *d_12_-PER* | NA^b^ |
| Acenaphthylene-*d8* | *d_8_*-ACY | 98.2 | Labor^l^ | NA^b^ | *d_12_-PER* | NA^b^ |
| Acenaphthene-*d10* | *d_10_*-ACE | 97.9 | Labor^l^ | NA^b^ | *d_12_-PER* | NA^b^ |
| Fluorene-*d10* | *d_10_*-FLO | 97.4 | Labor^l^ | NA^b^ | *d_12_-PER* | NA^b^ |
| Phenanthrene-*d10* | *d_10_*-PHE | 97.8 | Labor^l^ | NA^b^ | *d_12_-PER* | NA^b^ |
| Anthracene-*d10* | *d_10_*-ANT | 98.9 | Labor^l^ | NA^b^ | *d_12_-PER* | NA^b^ |
| Fluoranthene-*d10* | *d_10_*-FLT | 98 | Labor^l^ | NA^b^ | *d_12_-PER* | NA^b^ |
| Pyrene-*d10* | *d_10_*-PYR | 96.8 | Labor^l^ | NA^b^ | *d_12_-PER* | NA^b^ |
| Benzo[*a*]anthracene-*d12* | *d_12_*-BaA | 98.3 | Labor^l^ | NA^b^ | *d_12_-PER* | NA^b^ |
| Chrysene-*d12* | *d_12_*-CHR | 97.6 | Labor^l^ | NA^b^ | *d_12_-PER* | NA^b^ |
| Benzo[*b*]fluoranthene-*d12* | *d_12_*-BbF | 99.2 | Labor^l^ | NA^b^ | *d_12_-BeP* | NA^b^ |
| Benzo[*k*]fluoranthene-*d12* | *d_12_*-BkF | 98.9 | Labor^l^ | NA^b^ | *d_12_-BeP* | NA^b^ |
| Benzo[*a*]pyrene-*d12* | *d_12_*-BaP | 97.3 | Labor^l^ | NA^b^ | *d_12_-BeP* | NA^b^ |
| Benzo[*ghi*]perylene-*d12* | *d_12_*-BghiP | 97.5 | Labor^l^ | NA^b^ | *d_12_-BeP* | NA^b^ |
| Dibenzo[*a*,*h*]anthracene-*d14* | *d_14_*-DBahA | 99.1 | Labor^l^ | NA^b^ | *d_12_-BeP* | NA^b^ |
| Indeno[1,2,3-*cd*]pyrene-*d12* | *d_12_*-IcdP | 98 | Labor^l^ | NA^b^ | *d_12_-BeP* | NA^b^ |
| ***Alkyl-PAHs and PASHs IS*** |  |  |  |  |  |  |
| 9-methylanthracene-*d_12_* | *d_12_*-9-MANT | 98 | Chiron^k^ | NA^b^ | *d_12_-PER* | NA^b^ |
| 1‑methylnaphthalene-*d_10_* | *d_10_*-1-MNAP | 98.8 | Chiron^k^ | NA^b^ | *d_12_-PER* | NA^b^ |
| Dibenzothiophene-*d_8_* | *d_8_*-DBT | 98.7 | Chiron^k^ | NA^b^ | *d_12_-PER* | NA^b^ |
| 5-methlychrysene (methyl-*d_3_*) | *Md_3_-5MCHR* | 98 | LGC^e^ | NA^b^ | *d_12_-PER* | NA^b^ |
| ***OPAHs, azaarenes, and OPACs IS*** |  |  |  |  |  |  |
| Carbazole-*d_8_* | *d_8_*-CARB | 98.9 | Chiron^k^ | NA^b^ | *d_12_-PER* | NA^b^ |
| Acridine-*d_9_* | *d_9_*-ACR | 98.7 | Chiron^k^ | NA^b^ | *d_12_-PER* | NA^b^ |
| Anthraquinone-*d_8_* | *d_8_*-AQN | 98 | Chiron^k^ | NA^b^ | *d_12_-PER* | NA^b^ |
| ***RS*** |  |  |  |  |  |  |
| Perylene-*d_12_* | *d_12_*-PER | 98 | Sigma^g^ | NA^b^ | - | NA^b^ |
| Benzo[*e*]pyrene‑*d_12_* | *d_12_*-BeP | 98.4 | LGC^e^ | NA^b^ | - | NA^b^ |

^a^ Predicted using U.S. EPA CompTox dashboard (<https://comptox.epa.gov/dashboard/>)

^b^ Not applicable/available

^c^ SRM 2260a mixture from Sigma-Aldrich (Stockholm, Sweden)

^d^ Ultra Scientific Analytical Solutions (North Kingstown, USA)

^e^ LGC (Wesel, Germany)

^f^ S-4406-200-2 T mixture from Chiron AS (Trondheim, Norway)

^g^ Sigma-Aldrich (Stockholm, Sweden)

^h^ Alfa Aesar (Karlsruhe, Germany)

^i^ Fisher Scientific (Göteborg, Sweden)

^j^ Tokyo Chemical (Eschborn, Germany)

^k^ Chiron AS (Trondheim, Norway)

^l^ Labor Dr. Ehrenstrofer-Schäfers (Augsburg, Germany)

**Figure S1.** Mass defect suspect screening analysis workflow.


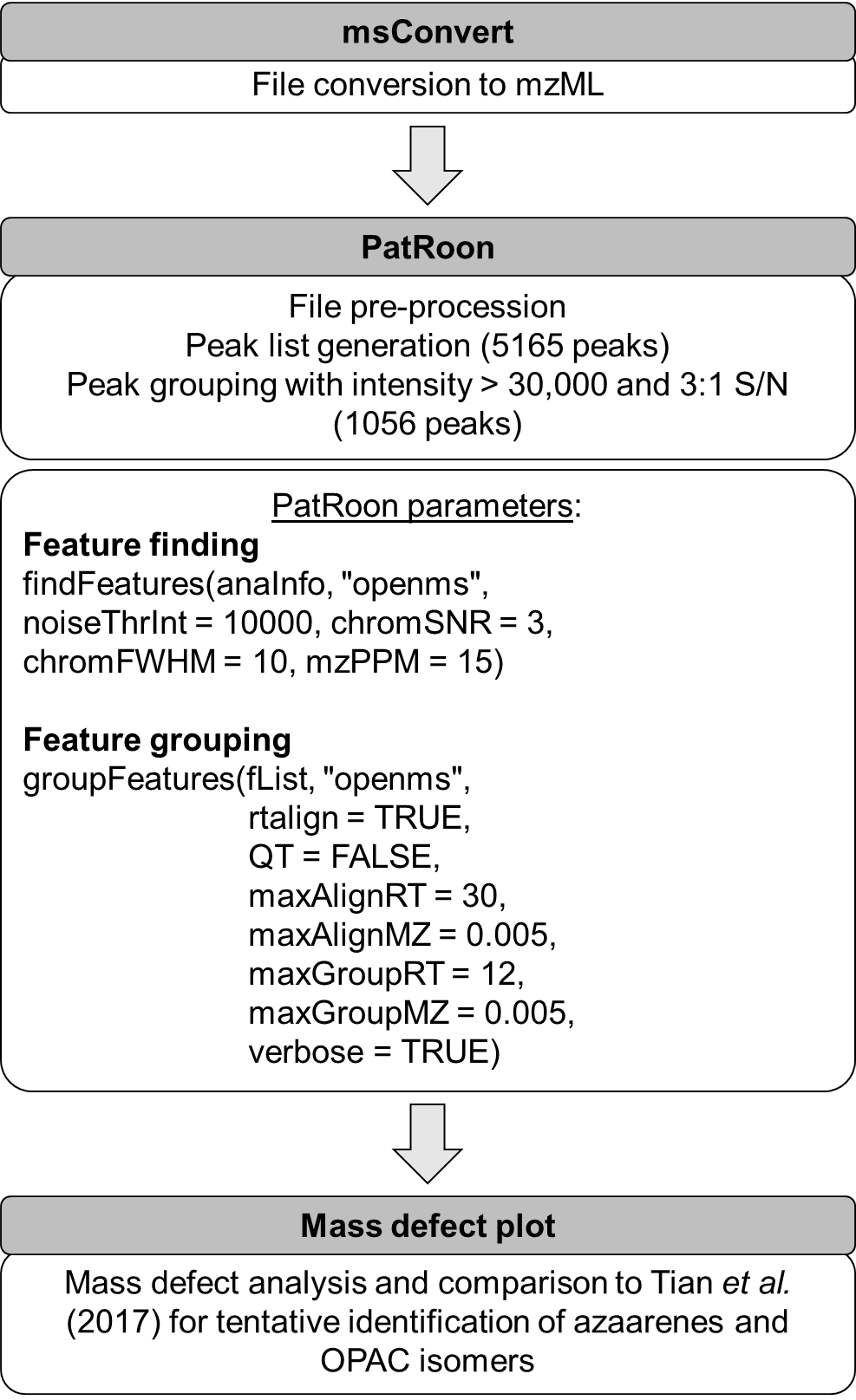


**Table S2.** List of log *K_POM_* for all PACs. Data were obtained from (Hawthorne et al., 2011) and (Josefsson et al., 2015) (for OPAHs).

| **Compound** | **Log K*_POM_*** | **Compound** | **Log K*_POM_*** | **Compound** | **Log K*_POM_*** |
| --- | --- | --- | --- | --- | --- |
| ***PAH 16*** | | ***Alkyl-PAHs*** | | ***Azaarenes*** | |
| NAP | 3.1 | 2-MNAP | 3.4 | QON* | 2.7 |
| ACY | 3.8 | 1-MNAP | 3.3 | BhQ* | 3.9 |
| ACE | 3.5 | 1,6-DMNAP | 3.6 | ACR* | 3.9 |
| FLO | 3.8 | 2,3,5-TMNAP | 3.6 | PDN* | 3.9 |
| PHE | 4.2 | 2-MPHE | 4.5 | CARB* | 3.6 |
| ANT | 4.3 | 2-MANT | 4.5 | 9-MACR* | 4.2 |
| FLT | 4.6 | 2,4-DMPHE | 4.9 | ACDN* | 3.9 |
| PYR | 4.6 | 2,3-DMANT | 4.9 | BaCARB* | 4.7 |
| BaA | 5.5 | 1,2,8-TMPHE | 5.3 | DBahACR* | 6.2 |
| CHR | 5.4 | 1,2,6-TMPHE | 5.3 | ***PASHs*** | |
| BbF | 5.8 | 1-MFLT | 4.9 | DBT* | 3.6 |
| BkF | 6.0 | 7-MBaA | 5.6 | 2-MDBT* | 3.9 |
| BaP | 5.7 | 3-MCHR | 5.6 | 2,8-DMBT* | 4.2 |
| IcdP | 6.3 | 2-MCHR | 5.6 | 2,4,7-TDMBT* | 4.5 |
| DBahA | 6.3 | 6-MCHR | 5.6 | ***OPACs*** | |
| BghiP | 6.1 | 5-MCHR | 5.6 | BNF* | 4.7 |
| ***Other parent-PAHs*** | | 1-MCHR | 5.6 | DNF* | 5.9 |
| BIP* | 3.6 | 6-ECHR* | 5.9 |  |  |
| CdP* | 4.5 | 7,12-DMBaA | 5.9 |  |  |
| BaFL* | 5.0 | 7-MBaP* | 6.2 |  |  |
| BcFL* | 5.0 | ***OPAHs*** | |  |  |
| BcP* | 5.3 | 1-IND | 1.0 |  |  |
| BghiF* | 5.3 | 9-FLN | 3.1 |  |  |
| CcdP* | 5.3 | XAN* | 3.9 |  |  |
| TRI* | 5.3 | ANQ+PHD (each) | 3.3 |  |  |
| BjF* | 5.9 | CdPN | 4.1 |  |  |
| BaF* | 5.9 | 2-MANQ | 3.9 |  |  |
| BeP | 6.0 | BaFLN | 5.2 |  |  |
| PER | 6.0 | BdeAON | 4.6 |  |  |
| DBajA* | 6.5 | BaAQN | 5.4 |  |  |
| DBacA* | 6.5 | CQN+NQN (each) | 5.2 |  |  |
| BbC* | 6.5 | BcdPN | 5.2 |  |  |
| PIC* | 6.5 | 9,10-DBaPN* | 5.9 |  |  |
| ANH* | 6.5 | *denotes PACs with predicted log *K_POM_* using equation from (Hawthorne et al., 2011) | | | |
| ***MW302-PAHs*** | |  |  |  |  |
| DBbkF* | 7.0 |  |  |  |  |
| DBaeP* | 7.0 |  |  |  |  |
| COR* | 7.0 |  |  |  |  |
| DBalP* | 7.0 |  |  |  |  |
| N23aP* | 7.0 |  |  |  |  |

**Table S3.** LOQs (ng g^-1^ d.m.) of PACs in soil.

| **Compound** | **LOQ** | **Compound** | **LOQ** | **Compound** | **LOQ** |
| --- | --- | --- | --- | --- | --- |
| ***PAH 16*** | | ***Alkyl-PAHs*** | | ***Azaarenes*** | |
| NAP | 33 | 2-MNAP | 5.9 | QON | 0.49 |
| ACY | 8.8 | 1-MNAP | 3.9 | BhQ | 1.8 |
| ACE | 8.8 | 1,6-DMNAP | 2.9 | ACR | 0.89 |
| FLO | 4.9 | 2,3,5-TMNAP | 2.0 | PDN | 1.8 |
| PHE | 42 | 2-MPHE | 54 | CARB | 0.89 |
| ANT | 3.9 | 2-MANT | 18 | 9-MACR | 2.7 |
| FLT | 13 | 2,4-DMPHE | 0.98 | ACDN | 0.78 |
| PYR | 12 | 2,3-DMANT | 0.98 | BaCARB | 0.89 |
| BaA | 3.9 | 1,2,8-TMPHE | 2.0 | DBahACR | 0.28 |
| CHR | 2.9 | 1,2,6-TMPHE | 2.0 | ***PASHs*** | |
| BbF | 0.98 | 1-MFLT | 2.0 | DBT | 0.98 |
| BkF | 2.0 | 7-MBaA | 0.98 | 2-MDBT | 0.49 |
| BaP | 3.9 | 3-MCHR | 0.98 | 2,8-DMBT | 0.98 |
| IcdP | 3.9 | 2-MCHR | 0.98 | 2,4,7-TDMBT | 0.49 |
| DBahA | 0.28 | 6-MCHR | 8.8 | ***OPACs*** | |
| BghiP | 2.0 | 5-MCHR | 6.9 | BNF | 0.14 |
| ***Other parent-PAHs*** | | 1-MCHR | 2.9 | DNF | 0.39 |
| BIP | 11 | 6-ECHR | 0.98 |  |  |
| CdP | 2.9 | 7,12-DMBaA | 36 |  |  |
| BaFL | 3.9 | 7-MBaP | 0.98 |  |  |
| BcFL | 0.98 | ***OPAHs*** | |  |  |
| BcP | 2.0 | 1-IND | 0.49 |  |  |
| BghiF | 0.98 | 9-FLN | 6.2 |  |  |
| CcdP | 2.0 | XAN | 11 |  |  |
| TRI | 0.32 | ANQ+PHD | 27 |  |  |
| BjF | 2.0 | CdPN | 0.89 |  |  |
| BaF | 3.9 | 2-MANQ | 0.89 |  |  |
| BeP | 2.9 | BaFLN | 0.89 |  |  |
| PER | 2.0 | BdeAON | 1.8 |  |  |
| DBajA | 0.34 | BaAQN | 2.7 |  |  |
| DBacA | 2.0 | CQN+NQN | 8.0 |  |  |
| BbC | 0.98 | BcdPN | 0.89 |  |  |
| PIC | 2.9 | 9,10-DBaPN | 2.7 |  |  |
| ANH | 2.0 |  |  |  |  |
| ***MW302-PAHs*** | |  |  |  |  |
| DBbkF | 6.9 |  |  |  |  |
| DBaeP | 6.9 |  |  |  |  |
| COR | 3.9 |  |  |  |  |
| DBalP | 0.98 |  |  |  |  |
| N23aP | 3.9 |  |  |  |  |

**Table S4.** LOQs (ng g^-1^ POM) of PACs in POM films.

| **Compound** | **LOQ** | **Compound** | **LOQ** | **Compound** | **LOQ** |
| --- | --- | --- | --- | --- | --- |
| ***PAH 16*** | | ***Alkyl-PAHs*** | | ***Azaarenes*** | |
| NAP | 280 | 2-MNAP | 13 | QON | 3.0 |
| ACY | 8.5 | 1-MNAP | 8.0 | BhQ | 1.0 |
| ACE | 63 | 1,6-DMNAP | 16 | ACR | 7.0 |
| FLO | 16 | 2,3,5-TMNAP | 5.0 | PDN | 10 |
| PHE | 260 | 2-MPHE | 25 | CARB | 8.0 |
| ANT | 6.4 | 2-MANT | 4.0 | 9-MACR | 2.0 |
| FLT | 75 | 2,4-DMPHE | 15 | ACDN | 2.0 |
| PYR | 63 | 2,3-DMANT | 8.0 | BaCARB | 3.0 |
| BaA | 5.0 | 1,2,8-TMPHE | 6.0 | DBahACR | 1.0 |
| CHR | 6.0 | 1,2,6-TMPHE | 2.0 | ***PASHs*** | |
| BbF | 10 | 1-MFLT | 2.0 | DBT | 5.0 |
| BkF | 1.0 | 7-MBaA | 0.49 | 2-MDBT | 1.0 |
| BaP | 2.0 | 3-MCHR | 1.0 | 2,8-DMDBT | 0.50 |
| IcdP | 1.0 | 2-MCHR | 2.0 | 2,4,7-TMDBT | 1.5 |
| DBahA | 0.47 | 6-MCHR | 9.1 | ***OPACs*** | |
| BghiP | 1.0 | 5-MCHR | 3.0 | BNF | 2.0 |
| ***Other parent-PAHs*** | | 1-MCHR | 2.0 | DNF | 0.48 |
| BIP | 34 | 6-ECHR | 1.0 |  |  |
| CdP | 16 | 7,12-DMBaA | 19 |  |  |
| BaFL | 5.0 | 7-MBaP | 2.0 |  |  |
| BcFL | 3.0 | ***OPAHs*** | |  |  |
| BcP | 3.0 | 1-IND | 8.0 |  |  |
| BghiF | 2.0 | 9-FLN | 33 |  |  |
| CcdP | 17 | XAN | 23 |  |  |
| TRI | 1.0 | ANQ+PHD | 32 |  |  |
| BjF | 2.0 | CdPN | 9.1 |  |  |
| BaF | 7.0 | 2-MANQ | 1.0 |  |  |
| BeP | 2.0 | BaFLN | 11 |  |  |
| PER | 1.0 | BdeAON | 3.0 |  |  |
| DBajA | 1.0 | BaAQN | 4.0 |  |  |
| DBacA | 14 | CQN+NQN | 6.0 |  |  |
| BbC | 1.0 | BcdPN | 4.0 |  |  |
| PIC | 5.0 | 9,10-DBaPN | 7.0 |  |  |
| ANH | 2.0 |  |  |  |  |
| ***MW302-PAHs*** | |  |  |  |  |
| DBbkF | 4.0 |  |  |  |  |
| DBaeP | 2.0 |  |  |  |  |
| COR | 1.0 |  |  |  |  |
| DBalP | 1.0 |  |  |  |  |
| N23aP | 2.0 |  |  |  |  |

**Table S5.** PAC concentrations in soil (ng g^-1^ d.m.) and in POM films (ng g^-1^ POM), and the freely dissolved concentrations (ng mL^-1^).

| **Compound** | **Concentrations in soil** | | | **Concentrations in POM films** | | | **Freely dissolved concentrations** | | |
| --- | --- | --- | --- | --- | --- | --- | --- | --- | --- |
|  | **Pre-SEE** | **Post-SEE** | **% change** | **Pre-SEE** | **Post-SEE** | **% change** | **Pre-SEE** | **Post-SEE** | **% change** |
| ***PAH 16*** |  |  |  |  |  |  |  | |  |
| NAP | 3,300 | 320 | -90 | 19,000 | 1,700 | -91 | 17 | 1.5 | -91 |
| ACY | 1,200 | 17 | -98 | 16,000 | 79 | -100 | 2.7 | 0.013 | -100 |
| ACE | 60,000 | 55 | -100 | 930,000 | 2,300 | -100 | 290 | 0.73 | -100 |
| FLO | 90,000 | 190 | -100 | 1,300,000 | 8,000 | -99 | 190 | 1.2 | -99 |
| PHE | 290,000 | 5,100 | -98 | 3,700,000 | 98,000 | -97 | 230 | 6.2 | -97 |
| ANT | 180,000 | 1,800 | -99 | 2,100,000 | 42,00 | -98 | 100 | 2.1 | -98 |
| FLT | 160,000 | 19,000 | -88 | 1,900,000 | 29,000 | -85 | 54 | 7.9 | -85 |
| PYR | 97,000 | 10,000 | -89 | 930,000 | 170,000 | -82 | 25 | 4.5 | -82 |
| BaA | 21,000 | 11,000 | -46 | 180,000 | 170,000 | -7.7 | 0.62 | 0.58 | -7.7 |
| CHR | 19,000 | 11,000 | -43 | 140,000 | 120,000 | -13 | 0.52 | 0.46 | -12 |
| BbF | 6,400 | 4,600 | -27 | 31,000 | 30,000 | -2.2 | 0.049 | 0.048 | -2.2 |
| BkF | 3,300 | 2,200 | -33 | 20,000 | 20,000 | -3.8 | 0.022 | 0.021 | -3.8 |
| BaP | 5,700 | 2,800 | -51 | 29,000 | 21,000 | -28 | 0.063 | 0.046 | -28 |
| IcdP | 1,800 | 1,300 | -29 | 7,100 | 4,300 | -31 | 0.0039 | 0.0027 | -31 |
| DBahA | 280 | 200 | -29 | 1,100 | 980 | -12 | 0.00056 | 0.00049 | -12 |
| BghiP | 1,100 | 800 | -28 | 3,300 | 1,900 | -44 | 0.0027 | 0.0015 | -44 |
| ∑_16 PAHs_ | 940,000 | 71,000 | -92 | 11,000,000 | 980,000 | -91 | 910 | 25 | -97 |
| ***Other parent-PAHs*** |  |  |  |  |  |  |  | |  |
| BIP | 820 | 17 | -98 | 6,300 | 78 | -99 | 1.6 | 0.020 | -99 |
| CdP | 30,000 | 420 | -99 | 240,000 | 8,900 | -96 | 8.5 | 0.31 | -96 |
| BaFL | 7,800 | 2,500 | -68 | 120,000 | 26,000 | -79 | 1.1 | 0.24 | -79 |
| BcFL | 8,800 | 850 | -90 | 22,000 | 2,100 | -91 | 0.21 | 0.020 | -91 |
| BcP | 3,900 | 1,700 | -58 | 29,000 | 21,000 | -28 | 0.14 | 0.10 | -28 |
| BghiF | 2,000 | 890 | -56 | 12,000 | 8,700 | -30 | 0.060 | 0.042 | -30 |
| CcdP | 9,100 | 5,000 | -45 | 80,000 | 74,000 | -8.4 | 0.39 | 0.36 | -8.4 |
| **Compound** | **Concentrations in soil** | | | **Concentrations in POM films** | | | **Freely dissolved concentrations** | | |
|  | **Pre-SEE** | **Post-SEE** | **% change** | **Pre-SEE** | **Post-SEE** | **% change** | **Pre-SEE** | **Post-SEE** | **% change** |
| TRI | 3,500 | 2,000 | -42 | 15,000 | 15,000 | -1.9 | 0.075 | 0.073 | -1.9 |
| BjF | 3,400 | 2,500 | -26 | 19,000 | 17,000 | -11 | 0.024 | 0.022 | -11 |
| BaF | 1,500 | 930 | -39 | 6,900 | 5,600 | -20 | 0.0088 | 0.0071 | -20 |
| BeP | 4,200 | 3,100 | -25 | 16,000 | 14,000 | -15 | 0.018 | 0.015 | -15 |
| PER | 1,300 | 570 | -57 | 5,600 | 2,800 | -50 | 0.0051 | 0.0026 | -50 |
| DBajA | 210 | 160 | -23 | 820 | 730 | -11 | 0.00028 | 0.00025 | -11 |
| DBacA | 310 | 240 | -24 | 1,500 | 1,100 | -29 | 0.00052 | 0.00037 | -29 |
| BbC | 340 | 240 | -31 | 1,900 | 1,600 | -17 | 0.00065 | 0.00054 | -17 |
| PIC | 610 | 480 | -22 | 3,400 | 2,100 | -36 | 0.0011 | 0.00073 | -36 |
| ANH | 240 | 47 | -80 | 860 | 200 | -77 | 0.00029 | 0.000067 | -77 |
| ∑_Other parent-PAHs_ | 78,000 | 22,000 | -72 | 580,000 | 200,000 | -66 | 12 | 1.2 | -90 |
| ***MW302-PAHs*** |  |  |  |  |  |  |  | |  |
| DBbkF | 230 | 150 | -35 | 490 | 240 | -51 | 0.000044 | 0.000022 | -51 |
| DBaeP | 140 | 100 | -30 | 440 | 240 | -46 | 0.000039 | 0.000021 | -46 |
| COR | 170 | 120 | -27 | 440 | 190 | -57 | 0.000040 | 0.000017 | -57 |
| DBalP | 150 | 110 | -27 | 360 | 140 | -60 | 0.000032 | 0.000013 | -60 |
| N23aP | <LOQ | <LOQ | NA | <LOQ | <LOQ | NA | <LOQ | <LOQ | NA |
| ∑_MW302-PAHs_ | 690 | 490 | -30 | 1,730 | 810 | -53 | 0.00016 | 0.000073 | -53 |
| ***Alkyl-PAHs*** |  |  |  |  |  |  |  | |  |
| 2-MNAP | 3,300 | 37 | -99 | 17,000 | 150 | -99 | 7.4 | 0.063 | -99 |
| 1-MNAP | 1,400 | 19 | -99 | 33,000 | 74 | -100 | 16 | 0.035 | -100 |
| 1,6-DMNAP | 16,000 | 32 | -100 | 260,000 | 580 | -100 | 66 | 0.15 | -100 |
| 2,3,5-TMNAP | 5,200 | 9.4 | -100 | 63,000 | 380 | -99 | 14 | 0.086 | -99 |
| 2-MPHE | 21,000 | 470 | -98 | 280,000 | 12,000 | -96 | 9.2 | 0.40 | -96 |
| 2-MANT | 16,000 | 300 | -98 | 140,000 | 5,500 | -96 | 4.5 | 0.18 | -96 |
| 2,4-DMPHE | 2,000 | 180 | -91 | 13,000 | 1,200 | -91 | 0.16 | 0.015 | -91 |
| 2,3-DMANT | 1,500 | 110 | -92 | 10,000 | 1,600 | -85 | 0.13 | 0.020 | -85 |
| 1,2,8-TMPHE | 600 | 47 | -92 | 3,300 | 1,000 | -68 | 0.016 | 0.0051 | -68 |
| 1,2,6-TMPHE | 410 | 53 | -87 | 2,100 | 980 | -53 | 0.010 | 0.0048 | -53 |
| 1-MFLT | 4,900 | 400 | -92 | 25,000 | 9,200 | -62 | 0.31 | 0.12 | -62 |
| **Compound** | **Concentrations in soil** | | | **Concentrations in POM films** | | | **Freely dissolved concentrations** | | |
|  | **Pre-SEE** | **Post-SEE** | **% change** | **Pre-SEE** | **Post-SEE** | **% change** | **Pre-SEE** | **Post-SEE** | **% change** |
| 7-MBaA | 390 | 44 | -89 | 3,000 | 2,500 | -17 | 0.0076 | 0.0062 | -17 |
| 3-MCHR | 590 | 360 | -39 | 4,600 | 4,400 | -3.9 | 0.011 | 0.011 | -3.9 |
| 2-MCHR | 1,100 | 690 | -36 | 9,300 | 9,400 | +0.81 | 0.023 | 0.023 | +0.81 |
| 6-MCHR | <LOQ | <LOQ | NA | <LOQ | <LOQ | NA | <LOQ | <LOQ | NA |
| 5-MCHR | 370 | 230 | -38 | 2,700 | 2,400 | -9.0 | 0.0067 | 0.0061 | -9.0 |
| 1-MCHR | 630 | 410 | -35 | 4,900 | 3,800 | -22 | 0.012 | 0.0095 | -22 |
| 6-ECHR | <LOQ | <LOQ | NA | <LOQ | <LOQ | NA | <LOQ | <LOQ | NA |
| 7,12-DMBaA | 2,000 | 1,900 | -1.2 | 2,700 | 2,300 | -15 | 0.0035 | 0.0030 | -15 |
| 7-MBaP | <LOQ | <LOQ | NA | <LOQ | <LOQ | NA | <LOQ | <LOQ | NA |
| ∑_Alkyl-PAHs_ | 77,000 | 5,300 | -93 | 860,000 | 58,000 | -93 | 120 | 7.5 | -94 |
| ***OPAHs*** |  |  |  |  |  |  |  | |  |
| 1-IND | 80 | 30 | -63 | 300 | 47 | -85 | 33 | 5.1 | -85 |
| 9-FLN | 1,500 | 1,700 | +11 | 49,000 | 14,000 | -72 | 36 | 10 | -72 |
| XAN | <LOQ | <LOQ | NA | <LOQ | <LOQ | NA | <LOQ | <LOQ | NA |
| AQN+PHD | 3,600 | 9,200 | +150 | 260,000 | 220,000 | -14 | 62 | 53 | -14 |
| CdPN | 1,100 | 5,000 | +370 | 130,000 | 85,000 | -32 | 9.7 | 6.6 | -32 |
| 2-MANQ | <LOQ | <LOQ | NA | <LOQ | <LOQ | NA | <LOQ | <LOQ | NA |
| BaFLN | 1,500 | 4,400 | +190 | 73,000 | 77,000 | +5.7 | 0.43 | 0.46 | +5.7 |
| BdeAON | 60 | 140 | +140 | 2,000 | 2,500 | +23 | 0.047 | 0.057 | +23 |
| BaAQN | 60 | 150 | +140 | 5,100 | 6,600 | +29 | 0.022 | 0.029 | +29 |
| CQN+NQN | 440 | 1,400 | +220 | 66,000 | 62,000 | -5.6 | 0.23 | 0.22 | -5.6 |
| BcdPN | <LOQ | <LOQ | NA | <LOQ | <LOQ | NA | <LOQ | <LOQ | NA |
| 9,10-DBaPN | <LOQ | <LOQ | NA | <LOQ | <LOQ | NA | <LOQ | <LOQ | NA |
| ∑_OPAHs_ | 8,400 | 22,000 | +160 | 580,000 | 470,000 | -19 | 160 | 93 | -42 |
| ***Azaarenes*** |  |  |  |  |  |  |  | |  |
| QON | <LOQ | <LOQ | NA | <LOQ | <LOQ | NA | <LOQ | <LOQ | NA |
| BhQ | 3,200 | 1,900 | -42 | 46,000 | 46,000 | -1.2 | 6.1 | 6.1 | -1.2 |
| ACR | 410 | 420 | +3.9 | 11,000 | 8,800 | -18 | 1.4 | 1.2 | -18 |
| PDN | <LOQ | <LOQ | NA | <LOQ | <LOQ | NA | <LOQ | <LOQ | NA |
| **Compound** | **Concentrations in soil** | | | **Concentrations in POM films** | | | **Freely dissolved concentrations** | | |
|  | **Pre-SEE** | **Post-SEE** | **% change** | **Pre-SEE** | **Post-SEE** | **% change** | **Pre-SEE** | **Post-SEE** | **% change** |
| CARB | 14,000 | 250 | -98 | 97,000 | 14,000 | -86 | 25 | 3.6 | -86 |
| 9-MACR | <LOQ | <LOQ | NA | <LOQ | <LOQ | NA | <LOQ | <LOQ | NA |
| ACDN | <LOQ | <LOQ | NA | <LOQ | <LOQ | NA | <LOQ | <LOQ | NA |
| BaCARB | 310 | 87 | -72 | 3,500 | 3,300 | -7.1 | 0.064 | 0.059 | -7.1 |
| DBahACR | 30 | 24 | -19 | 230 | 300 | +26 | 0.00015 | 0.00019 | +26 |
| ∑_Azaarenes_ | 18,000 | 2,700 | -85 | 160,000 | 72,000 | -54 | 43 | 21 | -50 |
| ***PASHs*** |  |  |  |  |  |  |  | |  |
| DBT | 5,700 | 94 | -98 | 76,000 | 2,200 | -97 | 19 | 0.57 | -97 |
| 2-MDBT | 1,200 | 19 | -98 | 8,500 | 360 | -96 | 1.1 | 0.048 | -96 |
| 2,8-DMDBT | 330 | <LOQ | -100 | 1,100 | 150 | -87 | 0.078 | 0.010 | -87 |
| 2,4,7-TMDBT | <LOQ | <LOQ | NA | <LOQ | <LOQ | NA | <LOQ | <LOQ | NA |
| ∑_PASHs_ | 7,200 | 110 | -98 | 85,000 | 2,700 | -97 | 21 | 0.63 | -97 |
| ***OPACs*** |  |  |  |  |  |  |  | |  |
| BNF | 4,400 | 650 | -85 | 37,000 | 9,900 | -73 | 0.67 | 0.18 | -73 |
| DNF | 77 | 57 | -26 | 450 | 500 | +11 | 0.00058 | 0.00064 | +11 |
| ∑_OPACs_ | 4,500 | 700 | -84 | 37,000 | 10,000 | -72 | 0.67 | 0.18 | -73 |
| ∑_PACs_ | 1,100,000 | 130,000 | -89 | 14,000,000 | 1,800,000 | -87 | 1,300 | 150 | -88 |

**Table S6.** Chem-TEQs of PACs with assigned REP values (Table S1) in the soil extracts (ng g^‑1^d.m.) based on the H4IIE-*luc* reporter bioassay. * denotes PACs with REP value of 0.

| **Compound** | **Pre-SEE** | **Post-SEE** | **% change** |
| --- | --- | --- | --- |
| ***PAH 16*** |  |  |  |
| NAP* | 0 | 0 | NA |
| ACY* | 0 | 0 | NA |
| ACE* | 0 | 0 | NA |
| FLO* | 0 | 0 | NA |
| PHE* | 0 | 0 | NA |
| ANT* | 0 | 0 | NA |
| FLT* | 0 | 0 | NA |
| PYR | 0.48 | 0.052 | -89 |
| BaA | 0.27 | 0.14 | -46 |
| CHR | 0.76 | 0.45 | -41 |
| BbF | 3.4 | 2.5 | -27 |
| BkF | 7.8 | 5.2 | -33 |
| BaP | 0.31 | 0.15 | -51 |
| IcdP | 0.74 | 0.53 | -29 |
| BghiP | 0.0029 | 0.0021 | -28 |
| DBahA | 0.41 | 0.29 | -29 |
| ∑_16 PAHs_ | 14 | 9.3 | -35 |
| ***Other parent-PAHs*** |  |  |  |
| CdP | 0.0087 | 0.00012 | -99 |
| BaFL | 0.12 | 0.038 | -68 |
| TRI | 0.0010 | 0.00059 | -42 |
| BjF | 1.4 | 1.0 | -26 |
| BeP | 0.0029 | 0.0022 | -25 |
| PER* | 0 | 0 | NA |
| DBajA | 0.12 | 0.095 | -23 |
| DBacA | 0.18 | 0.14 | -24 |
| ∑_Other PAHs_ | 1.8 | 1.3 | -29 |
| ***MW302-PAH*** |  |  |  |
| N23aP | <LOQ | <LOQ | NA |
| ***Alkyl-PAHs*** |  |  |  |
| 2-MPHE | 0.0031 | 0.000071 | -98 |
| 2-MANT | 0.0050 | 0.000097 | -98 |
| 2,4-DMPHE | 0.00027 | 0.000024 | -91 |
| 2,3-DMANT | 0.0016 | 0.00017 | -90 |
| 1,2,8-TMPHE | 0.00060 | 0.00011 | -82 |
| 1,2,6-TMPHE | 0.00038 | 0.00013 | -66 |
| 1-MFLT | 0.00084 | 0.00023 | -73 |
| 7-MBaA | 0.11 | 0.054 | -49 |
| 3-MCHR | 1.2 | 0.72 | -39 |
| 2-MCHR | 1.3 | 0.83 | -36 |
| 1-MCHR | 0.16 | 0.10 | -35 |
| 6-ECHR | <LOQ | <LOQ | NA |
| 7-MBaP | <LOQ | <LOQ | NA |
| ∑_Alkyl-PAHs_ | 2.7 | 1.7 | -38 |
| ***OPAHs*** |  |  |  |
| 9-FLN* | 0 | 0 | NA |
| AQN+PHD* | 0 | 0 | NA |
| CdPN* | 0 | 0 | NA |
| 2-MANQ | <LOQ | <LOQ | NA |
| BaFLN | 0.0026 | 0.0075 | +190 |
| BdeAON | 0.000037 | 0.000089 | +140 |
| BaAQN | 0.000090 | 0.00022 | +140 |
| CQN+NQN | 0.0018 | 0.0056 | +220 |
| BcdPN | <LOQ | <LOQ | NA |
| 9,10-DBaPN | <LOQ | <LOQ | NA |
| ∑_OPAHs_ | 0.0044 | 0.013 | +200 |
| ***Azaarenes*** |  |  |  |
| 1-IND* | 0 | 0 | NA |
| QON* | <LOQ | <LOQ | NA |
| BhQ* | 0 | 0 | NA |
| ACR | 0.000065 | 0.00068 | +3.9 |
| CARB* | 0 | 0 | NA |
| 9-MACR* | <LOQ | <LOQ | NA |
| ACDN* | <LOQ | <LOQ | NA |
| BaCARB* | 0 | 0 | NA |
| DBahACR | 0.11 | 0.11 | +4.9 |
| of∑_Azaarenes_ | 0.11 | 0.11 | +4.9 |
| ***PASHs*** |  |  |  |
| DBT | 0.00026 | 0.000012 | -95 |
| 2-MDBT | 0.000061 | 0.0000049 | -92 |
| 2,8-DMDBT | 0.00025 | <LOQ | -100 |
| ∑_PASHs_ | 0.00057 | 0.000017 | -97 |
| ***OPAC*** |  |  |  |
| BNF | 0.0030 | 0.00045 | -85 |
| ∑_PACs_ | 19 | 12 | -34 |

**Table S7.** Isomers of azaarenes and OPACs detected using the mass defect suspect screening approach.

| **Azaarenes and OPAC (example compound given)** | **Formula** | **Monoisotopic mass (Da)** | **No. of isomers decreased post-SEE** | **No. of isomers with no change post-SEE** | **No. of isomers increased post-SEE** |
| --- | --- | --- | --- | --- | --- |
| carbazole | C_12_H_9_N | 167.0735 | 2 | - | - |
| methyl-carbazole | C_13_H_11_N | 181.8910 | - | - | - |
| dimethyl-carbazole | C_14_H_13_N | 195.1048 | 6 | - | - |
| benzocarbazole | C_16_H_11_N | 217.0891 | 6 | 9 | - |
| phenanthridine | C_13_H_9_N | 179.0735 | 2 | 4 | 1 |
| methyl-phenanthridine | C_14_H_11_N | 193.0891 | 9 | 5 | - |
| dimethyl-phenanthridine | C_15_H_13_N | 207.1048 | 18 | 6 | - |
| trimethyl-phenanthridine | C_16_H_15_N | 221.1204 | 16 | 1 | - |
| phenylquinoline | C_15_H_11_N | 205.0891 | 6 | 1 | - |
| methyl-phenylquinoline | C_16_H_13_N | 219.1048 | 11 | 3 | - |
| dimethyl-phenylquinoline | C_17_H_15_N | 233.1204 | 10 | 4 | - |
| azapyrene | C_15_H_9_N | 203.0735 | 2 | 4 | - |
| methyl-azapyrene | C_16_H_11_N | 217.0891 | 6 | 9 | - |
| dimethyl-azapyrene | C_17_H_13_N | 231.1048 | 10 | 3 | - |
| trimethyl-azapyrene | C_18_H_15_N | 245.1204 | 2 | 1 | - |
| benzoacridine | C_17_H_11_N | 229.0891 | 4 | 6 | - |
| methyl-benzoacridine | C_18_H_13_N | 243.1048 | 6 | 9 | 2 |
| dimethyl-benzoacridine | C_19_H_15_N | 257.1204 | 10 | 10 | - |
| trimethyl-benzoacridine | C_20_H_17_N | 271.1361 | 1 | 2 | - |
| phenylacridine | C_19_H_13_N | 255.1048 | 1 | 3 | - |
| methyl-phenylacridine | C_20_H_15_N | 269.1204 | 2 | 1 | - |
| azabenzopyrene | C_19_H_11_N | 253.0891 | 6 | 10 | 1 |
| methyl-azabenzopyrene | C_20_H_13_N | 267.1048 | 4 | 8 | - |
| dimethyl-azabenzopyrene | C_21_H_15_N | 281.1204 | 3 | 1 | - |
| dibenzoacridine | C_21_H_13_N | 279.1048 | 2 | 8 | - |
| methyl-dibenzoacridine | C_22_H_15_N | 293.1204 | - | 7 | - |
| dimethyl-dibenzoacridine | C_23_H_17_N | 307.1361 | 1 | 4 | 1 |
| benzonaphthofuran | C_16_H_10_O | 218.0732 | 3 | - | - |

**References**

Hawthorne, S.B., Jonker, M.T.O., van der Heijden, S.A., Grabanski, C.B., Azzolina, N.A., Miller, D.J., 2011. Measuring Picogram per Liter Concentrations of Freely Dissolved Parent and Alkyl PAHs (PAH-34), Using Passive Sampling with Polyoxymethylene. Anal. Chem. 83, 6754–6761. https://doi.org/10.1021/ac201411v

Josefsson, S., Arp, H.P.H., Kleja, D.B., Enell, A., Lundstedt, S., 2015. Determination of polyoxymethylene (POM) – water partition coefficients for oxy-PAHs and PAHs. Chemosphere 119, 1268–1274. https://doi.org/10.1016/j.chemosphere.2014.09.102
